# Development of an In Vitro Whole Blood Model to Study Anti-Inflammatory Effects of Strawberry Polyphenolic Compounds and Postprandial Inflammation

**DOI:** 10.3390/mps9010023

**Published:** 2026-02-07

**Authors:** Skyelar Reuter, Peter Geevarghese Alex, Casey Weisfuss, Britt Burton-Freeman, Indika Edirisinghe

**Affiliations:** Center for Nutrition Research, Department of Food Science and Nutrition, Illinois Institute of Technology, Chicago, IL 60616, USA; sreuter@illinoistech.edu (S.R.); pgeevarghesealex@illinoistech.edu (P.G.A.); cweisfuss@illinoistech.edu (C.W.); bburton@illinoistech.edu (B.B.-F.)

**Keywords:** postprandial inflammation, polyphenols, anti-inflammatory, hyperglycemia

## Abstract

Postprandial inflammation, characterized by elevated cytokines, is linked to metabolic diseases. Polyphenol-rich fruits like strawberries possess anti-inflammatory properties that may help reduce this response. Because clinical studies are often costly and time-consuming, this study aimed to develop an in vitro model using whole blood to examine the effect of bioactive components such as polyphenols on postprandial inflammation. Whole blood from healthy adults was exposed to lipopolysaccharides (LPS) and high glucose concentrations (250/500 mg/dL), as well as strawberry extract (100 ng/mL). Cytokines (interleukin-6 and -1 beta; IL-6, IL-1β, and Tumor Necrosis Factor-alpha; TNF-α) were quantified using the Luminex multiplex assay. High glucose levels caused non-significant increases in IL-6 (*p* > 0.05), while strawberry extracts significantly reduced LPS-induced cytokines (*p* < 0.05). These findings demonstrate the potential of using whole blood in vitro systems to model inflammation and to explore the anti-inflammatory effects of fruit components such as polyphenols from strawberries.

## 1. Introduction

Chronic diseases are the leading cause of morbidity and mortality in the modern era, significantly impacting both individual health and global economies [1]. These diseases, such as diabetes, cancer, cardiovascular disease (CVD), and certain cancers, result in prolonged suffering and reduced quality of life, while also posing a financial burden on national economies. The World Health Organization (WHO) considers chronic diseases a major global health crisis because they are becoming more common in both developed and developing countries [2].

Among the etiological factors contributing to the development of chronic diseases, chronic low-grade inflammation plays a significant role [3]. Diet/dietary factors are known to be associated with chronic low-grade inflammation. Modern-day “Western” diets are generally characterized by high intakes of refined carbohydrates and saturated fats and low intakes of essential nutrients. These diets are often energy-dense and nutrient-poor [4]. Research suggests that the quality of diet significantly influences inflammatory processes in chronic disease development. Postprandial inflammation is an acute inflammation response triggered after eating a high-carbohydrate/fat meal and is often exaggerated in individuals with metabolic inflexibility, such as people with or at risk for type 2 diabetes and CVD [5]. This postprandial inflammation is associated with increased pro-inflammatory cytokines, including interleukins (ILs) and tumor necrosis factor-alpha (TNF-α) [6]. However, in individuals with metabolic inflexibility, the body’s ability to regulate these inflammatory responses is impaired.

Several studies, including those conducted by our research group, have shown that a high intake of carbohydrates and fats can trigger inflammatory responses by activating inflammatory pathways leading to increased cytokine production [6,7,8]. These cytokines may contribute to the onset and progression of these diseases by promoting insulin resistance and endothelial dysfunction [4]. Data from clinical studies suggest that dietary interventions with plant bioactive components can reduce postprandial inflammation [7,8,9]. Conducting clinical studies to explore the health-promoting properties of dietary components is inherently time-consuming and costly, but an essential investment in understanding their effects on human subjects. The objective of this study was to develop an in vitro model to investigate the effect of nutrients/bioactive compounds on postprandial inflammation using human whole blood. Whole blood contains immune cells, which can respond to external stimuli and simulate the inflammatory processes that occur in vivo [10]. Using molecular biology techniques such as cytokine assays, this model will enable a detailed elucidation of the molecular mechanisms underlying inflammatory responses. This approach offers a cost-effective way to study diet–inflammation interactions prior to and supporting traditional clinical trials.

## 2. Experimental Design

### 2.1. Ethics-Blood Collection from Healthy Participants

This study was conducted in accordance with the Declaration of Helsinki and approved by the IRB of Illinois Institute of Technology, Chicago, Illinois (Protocol #IRB-2025-32, initial approval date 27 September 2024). Healthy participants (*n* = 23, 18–65 years, Body Mass Index- BMI 18.5–24.9 kg/m^2^, fasting glucose ≤ 125 mg/dL) were recruited from the Chicago metropolitan area and provided with written informed consent before the start of any study procedures. Participants were screened for eligibility based on current medication use, metabolic disorders, and chronic inflammatory conditions. All procedures were performed at the Clinical Nutrition Research Center (CNRC), Illinois Institute of Technology, Chicago, IL 60616. Demographic information on the subjects is given in Table 1.

### 2.2. Whole Blood Incubated with High Glucose

To test the effects of high blood glucose on inflammation, fasting (overnight 10–12 h) whole blood was collected from healthy adults (*n* = 23) via venipuncture into 6 mL ethylenediaminetetraacetic acid (EDTA) tubes. Samples were immediately placed on ice and processed within 30 min of collection. EDTA-containing whole blood (6 mL) was incubated with lipopolysaccharide (LPS-final concentration—2.5 ng/mL, Cat #Invitrogen™ 00497603, Waltham, MA, USA) low glucose (final initial glucose ~250 mg/dL), high glucose (final glucose ~500 mg/dL), and phosphate-buffered saline control (PBS—20–40 μL) at 37 °C for 4 h. We assumed that the fasting blood sugar level of the subjects was approximately 90 mg/dL. Consequently, we used 20 μL and 40 μL of Ansyr^®^ II dextrose-50% concentrated solution to achieve low (final concentration of approximately 250 mg/dL) and high (final concentration of approximately 500 mg/dL) glucose levels in the whole blood tubes, respectively. The volumes of the solutions added to the blood tubes were matched across experiments using PBS. During incubation, tubes were gently inverted every 10–15 min to prevent separation. After 4 h, plasma was separated by centrifugation at 456× *g* for 10 min at room temperature and aliquoted into microtubes. Samples were stored at −80 °C until cytokine analysis. Interleukin-6 (IL-6) concentrations were measured using the Luminex xMAP System (Biotechne, cat. # FCSTM14-01) according to the manufacturer’s protocol.

### 2.3. Whole Blood Incubated with LPS and Strawberry Extracts

Strawberry extracts were prepared using freeze-dried strawberry powder provided by the California Strawberry Commission [7]. The powder was dissolved in sterile PBS, vortexed for 10 min, and centrifuged at 456× *g* for 10 min. Strawberry extracts were prepared freshly just before the experiment began. The resulting supernatant was used as the working extract. The chemical composition of the strawberry extracts is given in our previous publication [11].

To test the effects of strawberry extract on LPS-induced inflammation, whole blood was collected from healthy adults (*n* = 6) via venipuncture into 3 mL EDTA tubes. The blood tubes (3 mL) were incubated with LPS (final concentration 1 ng/mL), strawberry (final concentration—100 ng/mL), LPS + strawberry extracts (final concentrations 1 ng/mL + 100 ng/mL), and PBS at 37 °C for 4 h. The volume added was matched across the experiments (30 μL). During incubation, tubes were gently inverted every 10–15 min to prevent separation. After 4 h of incubation, plasma was separated by centrifugation at 456× *g* for 10 min at room temperature and aliquoted into microtubes. Samples were stored at −80 °C until cytokine analysis. Cytokine concentrations for IL-6, TNF-α, and interleukin-1 beta (IL-1β) were analyzed using the Luminex xMAP System (Biotechne Cat #FCSTM14-03) according to the manufacturer’s protocol.

### 2.4. Data Analysis

Inflammatory marker data were analyzed using a two-tailed, paired *t*-test (https://www.statskingdom.com/, accessed on 23 December 2025). Normality of the data was checked based on the Shapiro–Wilk Test (α = 0.05). Each analyte concentration was compared to its respective control (PBS). In the experiment with strawberry extract, comparisons were made between LPS-treated and LPS-treated with strawberry extract to assess the modulatory effect of strawberry extract. Cytokine concentrations were expressed as mean ± standard deviation. Statistical significance determined as *p* < 0.05. All data were included in the analysis.

## 3. Results

**Figure 1:**
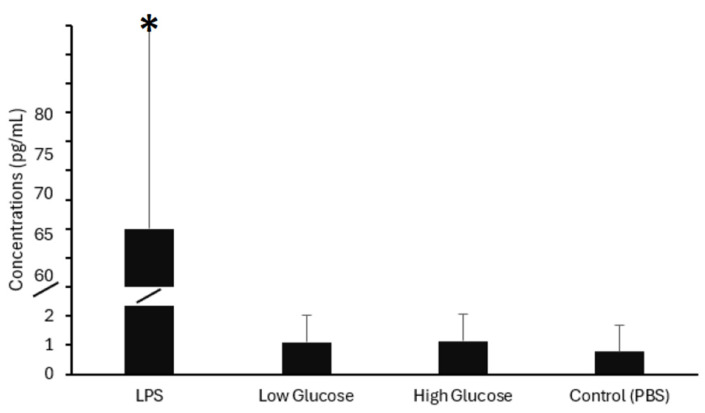
Effect of Lipopolysaccharides (LPS) and high glucose treatment on Interleukin-6 (IL-6) concentrations. Whole blood samples from human volunteers were incubated for 4 h at 37 °C with LPS (2.5 ng/mL), low glucose (250 mg/dL), high glucose (500 mg/dL), and phosphate-buffered saline (PBS). IL-6 levels were significantly higher in the LPS group than in the PBS control. * *p* < 0.05, *n* = 23.

As shown in Figure 1, incubation of whole blood samples from healthy individuals with LPS resulted in a significant increase in IL-6 secretion compared with the PBS control group (*p* < 0.05). Exposure to both low and high glucose concentrations resulted in a modest, non-significant increase in IL-6 levels compared to the PBS control (*p* > 0.05). Notably, incubation with 2.5 ng/mL of LPS resulted in a significantly higher IL-6 response, with concentrations reaching 60.93 ± 83.83 pg/mL, compared to 0.78 ± 0.90 pg/mL in the PBS control. Given this pronounced increase, we selected a lower LPS concentration (1 ng/mL) for subsequent experiments to better capture modulatory effects and avoid potential overstimulation of the inflammatory response.

**Figure 2:**
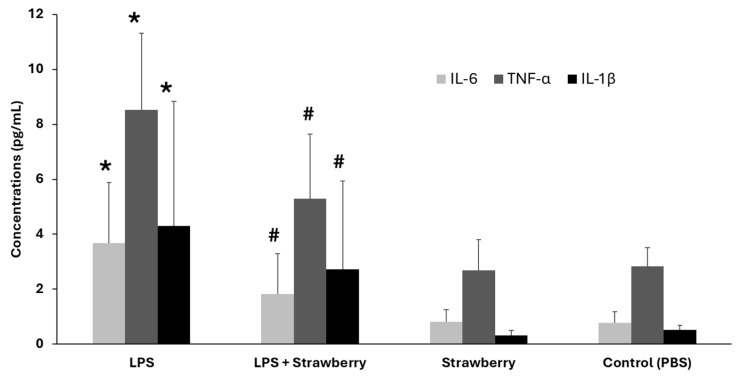
Effect of strawberry extract on lipopolysaccharides (LPS)-induced cytokine concentrations. Whole blood samples from human volunteers were incubated for 4 h at 37 °C with LPS (1 ng/mL), Strawberry extracts (100 ng/mL), LPS + strawberry extract, and phosphate-buffered saline (PBS). Interleukin-6 (IL-6), Tumor necrosis factor alpha (TNF-α) and Interleukin-1 Beta (IL-1β) were significantly increased in the LPS group compared to the PBS control * *p* < 0.05. Treatment with LPS and strawberry extracts significantly reduced LPS-induced IL-6, TNF-α, and IL-1β concentrations (# *p* < 0.05; *n* = 6).

In the next series of experiments, whole blood samples from healthy individuals were treated with strawberry extract (100 ng/mL) in the presence or absence of LPS (1 ng/mL) to evaluate the anti-inflammatory potential of water-soluble strawberry compounds. As shown in Figure 2, co-incubation with strawberry extract significantly attenuated the LPS-induced increased concentrations of IL-6, TNF-α, and IL-1β (*p* < 0.05). Treatment with the strawberry extract alone did not alter the levels of these inflammatory markers compared to the control (*p* > 0.05), indicating that the extract itself does not trigger an inflammatory response. Furthermore, no visible hemolysis or cell lysis was observed under any experimental condition, suggesting that the treatments were non-cytotoxic and that the results reflect modulatory effects on cytokine secretion.

## 4. Discussion

The present in vitro study used whole blood samples as a model to investigate inflammation. Our data indicated that LPS, a well-known inflammatory stimulus, significantly increased the release of inflammatory cytokines during the four-hour incubation period. The incubation duration was chosen because glucose spikes from high-carbohydrate meals typically occur within 30 to 60 min after ingestion, followed by cytokine release a few hours later [12]. This study used EDTA-anticoagulated blood, which may interfere with the detection of inflammatory stimuli due to its chelating effects. Although LPS still induced cytokine release within 4 h, future studies should use alternative anticoagulants with minimal impact on inflammatory responses to better assess inflammation [13,14].

In our experiments with glucose, in vitro exposure of whole blood to low- and high-glucose conditions did not result in significant increases in IL-6 levels, although a trend toward increased IL-6 was observed. This may be attributed to the use of whole blood samples from metabolically healthy individuals, which appeared capable of controlling elevated glucose concentrations without significantly affecting the inflammatory pathways. This warrants further investigation, including timing, to ensure a comprehensive understanding.

Clinical and experimental evidence from our laboratory and others has consistently demonstrated that polyphenolic compounds present in strawberries and other foods exhibit potent anti-inflammatory properties [15,16,17]. Several in vitro cell culture studies have confirmed that strawberry extracts can modulate key inflammatory pathways, suppressing cytokine production and oxidative stress [18]. Our previous work has shown that berry fruits, including strawberries, significantly attenuate endothelial dysfunction induced by high glucose and free fatty acids in human umbilical vein endothelial cells (HUVECs) [19]. These findings support the hypothesis that strawberry-derived polyphenolic compounds may help to reduce inflammation under metabolic stress conditions.

In the current in vitro whole blood model, we further demonstrated that strawberry extracts effectively reduced the LPS-induced release of proinflammatory cytokines, including IL-6, TNF-α, and IL-1β. Because LPS is a well-established activator of inflammatory signaling cascades through Toll-like receptor 4 (TLR4), our results suggest that components in strawberries can interfere with early inflammatory signaling, potentially by modulating NF-κB pathways [20].

Strawberries are particularly rich in anthocyanins, primarily pelargonidin and its glycosides, which are absorbed and detected in human plasma following consumption. These compounds appear both as parent molecules and metabolites, often conjugated as glucuronides or sulfates, with circulating concentrations typically in the nanomolar range [7]. In the present experimental design, we selected a strawberry extract concentration of 100 ng/mL, which corresponds to physiologically relevant levels of anthocyanin metabolites observed in vivo. This approach increases the translational relevance of our findings to human dietary intake.

Our previous clinical study in obese and insulin-resistant adults showed that regular strawberry intake significantly reduced postprandial insulin demand without adversely affecting glycemic control. Improvement in postprandial glycemia was correlated with circulating anthocyanin and pelargonidin metabolite concentrations [8]. Together, these findings support the notion that strawberry consumption can beneficially influence metabolic and inflammatory homeostasis, likely through modulation of postprandial oxidative and inflammatory responses. While the composition of extracts in the in vitro model does not fully mimic the complex metabolism of strawberry compounds following ingestion, the data presented here provide valuable mechanistic insight into the biological effects of strawberry-derived polyphenols. Specifically, our results indicate that physiologically relevant concentrations of strawberry compounds can directly suppress the production of inflammatory cytokines in human blood, underscoring their potential role in mitigating chronic, low-grade inflammation associated with metabolic disorders.

Overall, our findings strengthen the evidence that strawberry polyphenols, particularly anthocyanins, exert measurable anti-inflammatory effects at concentrations achievable through dietary intake. This in vitro model may serve as a useful tool to further explore the cellular and molecular mechanisms through which plant-derived compounds modulate postprandial inflammation. Furthermore, the magnitude and direction of these responses may differ among at-risk populations, such as individuals with prediabetes, obesity, or chronic low-grade inflammation, where baseline immune activation and metabolic dysfunction are altered. Future in vitro studies incorporating whole blood samples from such populations would be valuable for determining whether similar anti-inflammatory effects occur and for identifying interindividual variability in responsiveness to strawberry bioactive components. Such research could ultimately inform personalized nutrition strategies to reduce inflammation and improve metabolic health outcomes.

## 5. Conclusions

This study examined the anti-inflammatory effects of strawberry polyphenolic compounds using an in vitro whole blood model. While incubation with high glucose did not significantly increase inflammatory markers, strawberry polyphenols reduced LPS-induced inflammatory responses. The model offers a alternative way to study diet–inflammation interactions, though the findings cannot be directly translated to physiological effects in humans.

## Figures and Tables

**Table 1:** Participant demographic/baseline glucose information.

Variable	Values
Age (Years)	40 ± 16
Sex (Male/Female)	9/14
Race/Ethnicity	
African American	4
Asian	7
Hispanic	2
White	10
Fasting finger prick Glucose (mg/dL)	96.8 ± 7.8

## Data Availability

The data will be made available upon request.

## Abbreviations

The following abbreviations are used in this manuscript:

LPS	Lipopolysaccharides
STR	Strawberry
IL-6	Interleukin-6
IL-1β	Interleukin-1 beta
TNF-α	Tumor Necrosis Factor-alpha
CVD	Cardiovascular Diseases
WHO	World Health Organization
BMI	Body Mass Index
EDTA	Ethylenediaminetetraacetic Acid
PBS	Phosphate-Buffered Saline
